# Comprehensive Analysis of Prognostic Value and Immune Infiltration of MMP12 in Esophageal Squamous Cell Carcinoma

**DOI:** 10.1155/2022/4097428

**Published:** 2022-02-27

**Authors:** Jing-tao Mao, Qiang Lu, Peng-yu Jing, Zi-liang Li, Xiao-qi Yang, Ji-peng Zhang, Zhe Li

**Affiliations:** ^1^Department of Thoracic Surgery, Shaanxi Provincial Sengong Hospital, Xi'an, Shaanxi 710300, China; ^2^Department of Thoracic Surgery, Tangdu Hospital, The Air Force Military Medical University, Xi'an, Shaanxi 710038, China; ^3^Department of Thoracic Surgery, The Ninth Hospital of Xi' an, Xi'an, Shaanxi 710054, China

## Abstract

Esophageal squamous cell carcinoma (ESCC) is a typical neoplastic disease and a frequent cause of death in China. The prognosis of most ESCC patients is still poor. Previous studies demonstrated that MMP12 is involved in tumor metastasis. However, its clinical significance and association with cancer immunity remained largely unclear. In this study, we first analyzed the expressing pattern of MMPs in ESCC from TCGA datasets and found that several MMPs expression was distinctly increased in ESCC. However, only MMP12 expression was associated with five-year survival of ESCC patients. Then, we focused on MMP12 and found its high expression was positively related to advanced clinical stages of ESCC specimens. KEGG assays revealed MMP12 may influence the activity of several tumor-related pathways, such as the Toll-like receptor signaling pathway, TNF signaling pathway, and IL-17 signaling pathway. Then, we sought to determine whether MMP12 expressions were related to immune cell infiltration in ESCC. We observed that increased MMP12 levels were positively associated with the infiltration levels of mast cells activated and macrophages M0. However, eosinophils, B cells naïve, and mast cells resting exhibited an opposite result. Finally, we showed that knockdown of MMP12 suppressed the proliferation of ESCC cells. Overall, our findings proved that high expression of MMP12 may be a novel and valuable prognostic factor in ESCC.

## 1. Introduction

Esophageal squamous cell carcinoma (ESCC) remains the most prevailing histological subtype of esophageal cancer in developing nations or regions, such as China and Iran [1]. Among many types of clinical features associated with ESCC progression, distant metastases remain the critical element for unfavorable survivals [2]. Although the significant progresses have been achieved in the effective treatments of ESCC by the use of chemoradiotherapy and surgery in recent years, the 5-year survival rate remains <40% [3, 4]. Consequently, it is urgent to comprehend the genetic and molecular mechanism of ESCC to develop potential diagnostic therapy and treatment on ESCC.

As a family of zinc-dependent proteolytic enzymes, the matrix metalloproteinases (MMPs) are able to degrade the extracellular matrix and basement membrane [5]. More and more studies have demonstrated the positive effects of MMPs on the tumor grow, neoangiogenesis, migration, and metastasis [6, 7]. In the past two decades, several suppressors of MMPs have been developed in many types of tumors [8, 9]. However, although in vitro and in vivo experiments are very beneficial, the clinical experiments failed due to the lack of susceptibility and serious adverse reactions. Many researchers have analyzed the possible reason, and several MMPs which exhibit tumor-suppressor functions may be the most important one [10, 11]. With the developments of understating the potential function of MMPs in tumor progression, the sensitive narrow-spectrum MMPs inhibitors were currently being developed. In addition, some studies have reported the dysregulation of MMPs and their association with clinical outcome in several types of tumors [12–14].

In recent years, the effect of MMP12 has been verified in tumors. For example, MMP12 was highly expressed in adamantinomatous craniopharyngioma, while its knockdown inhibited the proliferation and attack of adamantinomatous craniopharyngioma cells [15]. Lin et al. reported that MMP12 was overexpressed in cervical cancer cells, and its silence clearly inhibited cell migration and invasion both in vitro and in vivo [16]. High expressions of MMP12 were related to the prognosis of several types of tumors, such as hepatocellular carcinoma and cutaneous melanoma [17, 18]. However, the expression and function of MMP12 in ESCC were rarely reported.

This study is to sort out the clinical significance of MMP12 on ESCC and its contribution to cancer immunity.

## 2. Methods and Materials

### 2.1. Data Collection from the TCGA Database

The data of RNA transcriptome and the corresponding clinicopathological and survival for patients were obtained from the Cancer Genome Atlas (TCGA, https://cancergenome.nih.gov/). All assays were carried out based on the publication guidelines of TCGA. 160 ESCC samples and 11 nontumor samples were enrolled in this study.

### 2.2. Cell Culture and Transfection

Het-1A and ESCC cell lines (KYSE30, EC-1, Eca109 and EC9706) were bought from the Shanghai Institute of Biochemistry and Cell Biology (Shanghai, China). The cells were cultured in RPMI-1640 medium supplemented with 10% fetal bovine serum (FBS) (Gibco BRL, USA) and maintained in a humidified incubator at 37°C with 5% CO_2_.

MMP12 small interfering RNA (si-MMP12) and the corresponding control RNA (si-NC) were purchased from Jinlai Biology (Beijing, China). Lipofectamine 3000 was applied for cellular transfection.

### 2.3. Identification of Differently Expressed MMPs

“Limma” package of R was applied to sort out the differently expressed MMPs between ESCC specimens and nontumor specimens. The keys with |logFC| ≥ 1 and *p* value <0.05 were defined as significant cutoff points. In addition, gene annotation and its data files of the differently expressed MMPs were collected through R software.

### 2.4. Prognosis-Related MMPs Screening

We constructed the Kaplan–Meier plots of MMPs in the TCGA dataset to comprehend the overall survival (OS) and verified it by log-rank tests.

### 2.5. Screening of Dysregulated Genes and GO and KEGG Pathway Assays

We carried out GO and KEGG pathway assays on the dysregulated genes between high MMP12 expression group and low MMP12 expression group: GO assays included molecular function (MF), cell component (CC), and biological process (BP). KEGG (http://www.genome.jp/) was a novel method for exploring the related regulatory pathways involved in gene functions. ClusterProfiler package was applied for GO and KEGG pathway assays, while GOplot package was applied for cluster assays [19]. Besides, it was thought to grind a significant difference when both the *p* value and *q* value were less than 0.05 only.

### 2.6. Assessment of Immune Infiltration

As a deconvolution algorithm, CIBERSORT applied the expressions of 547 tag genes to define the structure of immune cells in specimens. Hence, the associated proportion of 22 infiltrating immune cells was examined by the use of CIBERSORT in all samples from TCGA datasets. *P* <  0.05 was deem as statistically valuable.

### 2.7. Quantitative Real-Time PCR

RNA was isolated using TRIzol (Invitrogen, Pudong, Shanghai, China) following the manufacture's protocols. A Transcript RT kit (Vazyme, Nanjing, Jiangsu, China) was applied to compound the first strand cDNA. Real-time RT-PCR was performed to detect the expression of CRNDE using the One-Step SYBR PrimeScript RT-PCR Kit (Takara). GADPH was used as endogenous controls. The relative expressions were calculated using the 2^−ΔΔCt^ method.

### 2.8. CCK-8 Assays

Cellular proliferation was examined applying the Cell Counting Kit-8 (Beyotime, Haidian, China). Cells were seeded into 48-well plates at 3 × 10^3^ cells/well cell concentration. Then, 15 *μ*L CCK-8 solution was added to each well. At a wavelength of 450 nm for each well, the absorbance was examined.

### 2.9. Statistical Analysis

We adopted R (version 3.6.0) to conduct statistical analyses. The Wilcox test was applied to determine the dysregulated genes and infiltrative immune cells. We obtain the survival curves by the Kaplan–Meier method and compared by the log-rank test. *P* value <0.05 was considered statistically significant.

## 3. Results

### 3.1. Identification of the Dysregulated MMPs in ESCC

**Figure 1:**
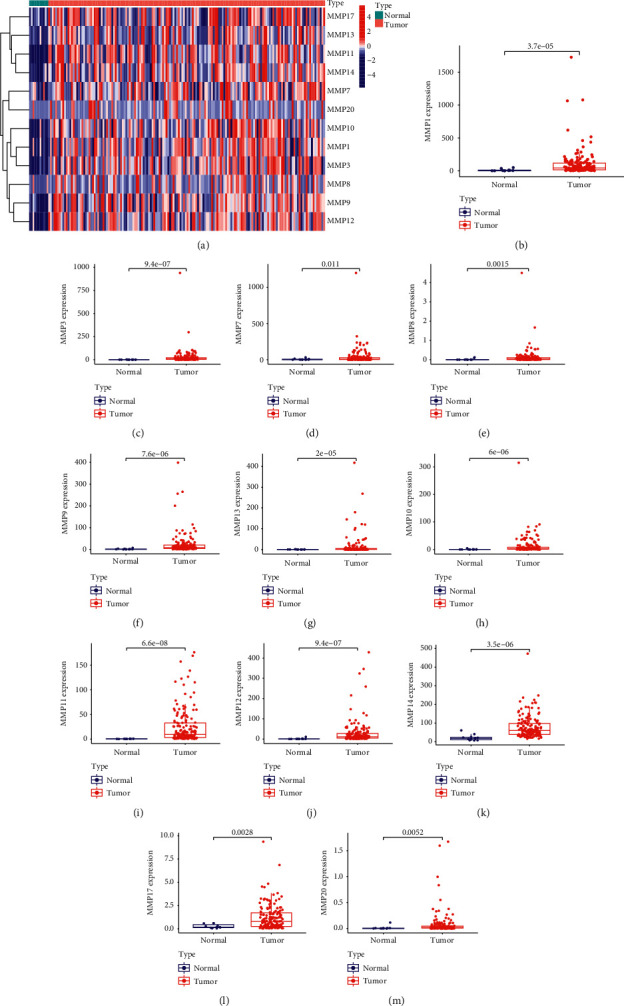
Identification of the dysregulated MMPs in ESCC. (a) Hierarchical clustering analysis of differently expressed MMPs in ESCC and normal tissues. (b)–(m) An increased expression of (b) MMP1, (c) MMP3, (d) MMP7, (e) MMP8, (f) MMP9, (g) MMP13, (h) MMP10, (i) MMP11, (j) MMP12, (k) MMP14, (l) MMP17, and (m) MMP20 observed in ESCC specimens compared with nontumor specimens.

To screen the dysregulated MMPs in ESCC, we analyzed TCGA datasets using Limma and edgeR packages. The dysregulated MMPs were shown in heat map (Figure 1(a)). We noticed that the behaviors of MMP12 were distinctly enhanced on ESCC specimens comparing to nontumor specimens, including MMP1 (Figure 1(b)), MMP3 (Figure 1(c)), MMP7 (Figure 1(d)), MMP8 (Figure 1(e)), MMP9 (Figure 1(f)), MMP13 (Figure 1(g)), MMP10 (Figure 1(h)), MMP11 (Figure 1(i)), MMP12 (Figure 1(j)), MMP14 (Figure 1(k)), MMP17 (Figure 1(l)), and MMP20 (Figure 1(m)).

### 3.2. The Survival-Related MMPs in ESCC

**Figure 2:**
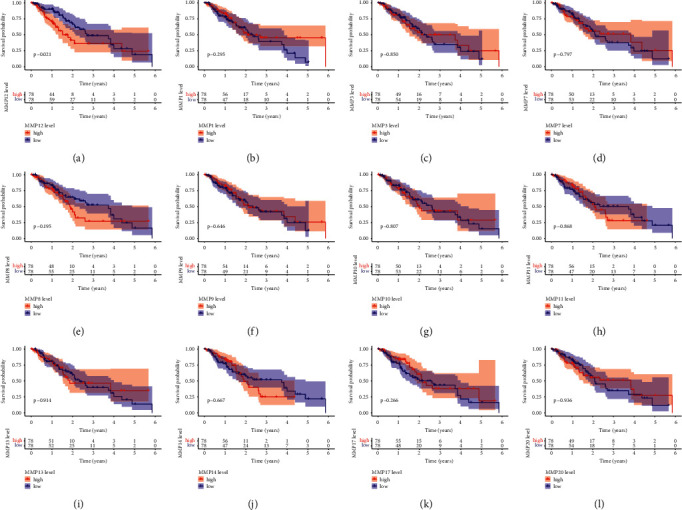
Identification of survival-related MMPs. (a) Survival analysis for MMP12 in ESCC. Patient with high MMP12 expression showed a shorter overall survival of ESCC patients. (b)–(l) Survival analysis for (b) MMP1, (c) MMP3, (d) MMP7, (e) MMP8, (f) MMP9, (g) MMP10, (h) MMP11, (i) MMP13, (j) MMP14, (k) MMP17, and (l) MMP20 in ESCC. According to median expression of MMPs, the patients were classified into high-level and low-level groups.

Then, we performed Kaplan–Meier methods to screen survival-related MMPs in ESCC. Only high MMP12 expression was associated with a short overall survival of ESCC patients (Figure 2(a)). For other MMPs, the results indicated no obvious difference in the survival rate between patients with high MMPs and low ones (Figures 2(b)–2(l)). Thus, our attention focused on MMP12.

### 3.3. Correlation between MMP12 Behaviors and Clinical Trials in ESCC Patients

**Figure 3:**
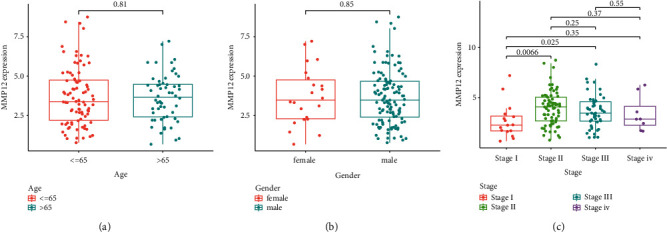
The relationship between MMP12 and clinical features. (a) Age (*p*= 0.81). (b) Gender (*p*= 0.85). (c) Clinical phase (*p* <  0.05).

We further examined the associations between MMP12 patients' clinical features and the MMP12 expressions and concluded that MMP12 expressions were not associated with age (*p*= 0.81, Figure 3(a)) and gender (*p*= 0.85, Figure 3(b)). However, we observed that the expressions of MMP12 in ESCC were distinctly linked to stage (Figure 3(c)).

### 3.4. Functional Enrichment Analysis of Genes That Were Coexpressed with MMP12

**Figure 4:**
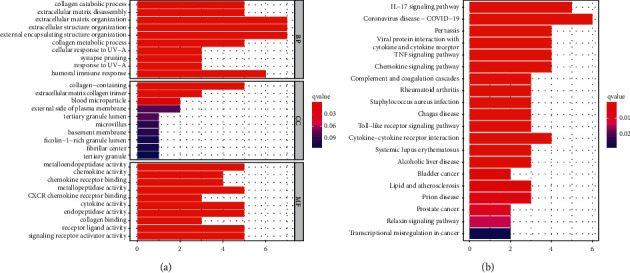
Functional enrichment analysis of the dysregulated genes in low and high MMP12 expression groups. (a) The enriched GO annotation included the biological process, cellular component, and molecular function. (b) Dotplot of the KEGG signal pathway.

To explore the biological function of MMP12 in ESCC, we divided all ESCC specimens into two (high and low) based on the mean expression of MMP12 in all ESCC samples. A total of 15 differently expressed genes between low and high MMP12 expression groups were screened. Next, 15 genes were chosen to perform GO and KEGG analyses using the ClusterProfiler R package. The results showed that MMP12-associated dysregulated genes were mainly involved in processes like the collagen catabolic process, extracellular matrix disassembly, collagen-containing, blood microparticle, metalloendopeptidase activity, and chemokine activity (Figure 4(a)). Meanwhile, KEGG pathway analysis showed that pathways were significantly enriched (Figure 4(b)) including the relaxin signaling pathway, Toll-like receptor signaling pathway, TNF signaling pathway, IL-17 signaling pathway, and transcriptional misregulation in cancer [20–22].

### 3.5. Distribution of Tumor-Infiltrating Immune Cells

**Figure 5:**
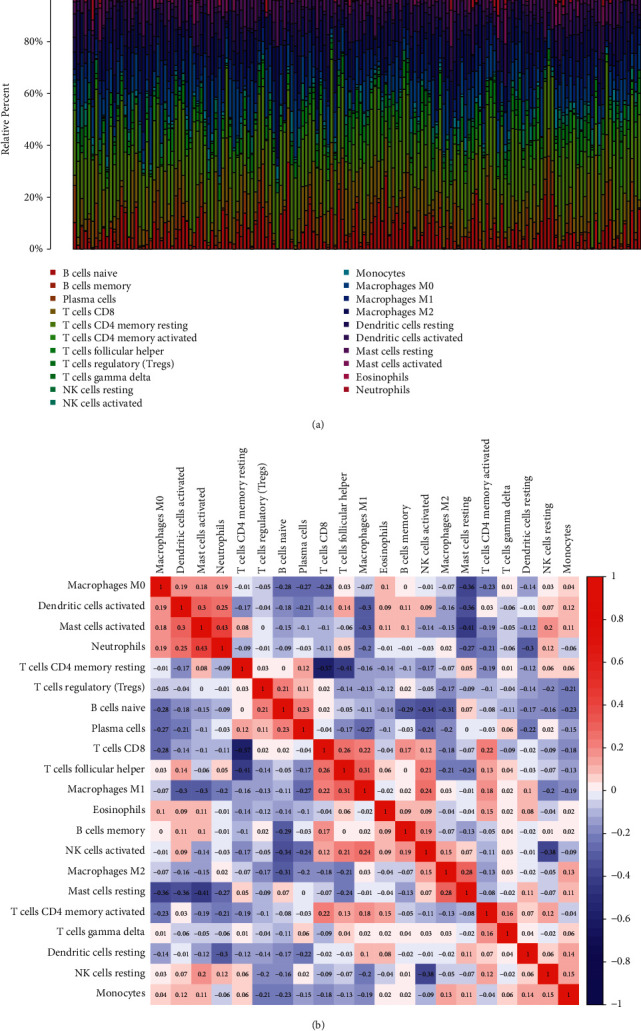
(a), (b) The proportion of the 22 immune cells detected by the CIBERSORT algorithm.

**Figure 6:**
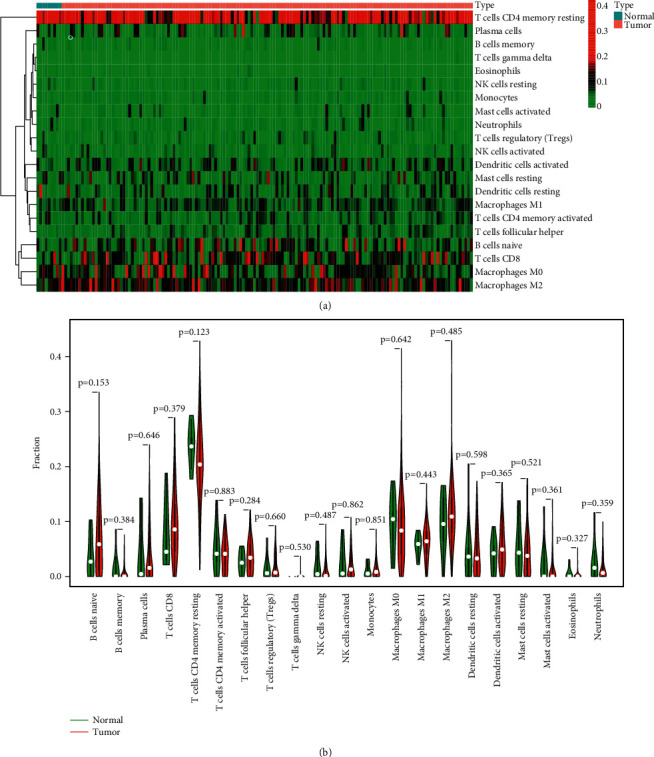
Analysis of the levels of 22 TIICs and its correlations in ESCC specimens and 11 normal cases. (a) Heatmaps indicated the expressing pattern of the immune cell between ESCC specimens and nontumor specimens. (b) The differences in the structure of TIICs between normal tissue and ESCC tissues.

**Figure 7:**
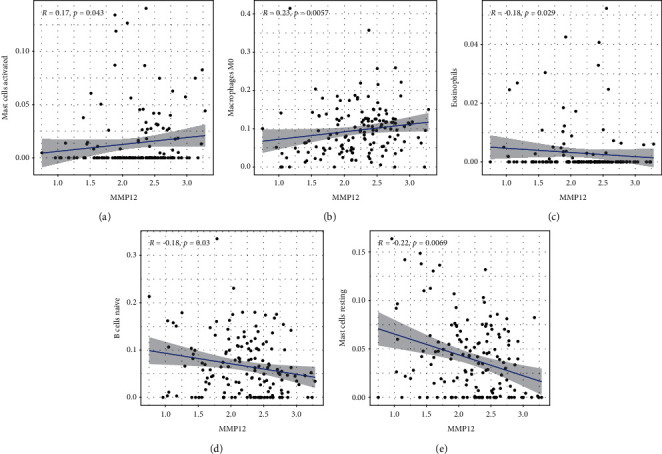
The correlation between MMP12 and immune infiltration level in ESCC. (a), (b) A positive association between MMP12 behaviors and the levels of macrophages M0 and mast cells activated was observed. (c)–(e) A negative association between MMP12 expression and the levels of B cells naïve, eosinophils, and mast cells resting was observed.

We explored the pattern of immune cells by the use of the CIBERSORT method. Its composition on ESCC samples and the associations among immune cells are shown in Figures 5(a) and 5(b), respectively. However, we found that there were no significant differences in the levels of tumor-infiltrating immune cells between tumor and nontumor specimens (Figures 6(a) and 6(b)). Several studies had proved that immune cells might serve as independent indicators of survivals and immunotherapy efficacies in ESCC [23, 24]. Then, we needed to finalize whether MMP12 behaviors were associated with immune cells. Importantly, we observed that the levels of MMP12 were in positive association with the infiltrated levels of mast cells activated (Figure 7(a)) and macrophages M0 (Figure 7(b)). However, eosinophils (Figure 7(c)), B cells naïve (Figure 7(d)), and mast cells resting (Figure 7(e)) exhibited an opposite result.

### 3.6. The Oncogenic Roles of MMP12 in ESCC Progression

**Figure 8:**
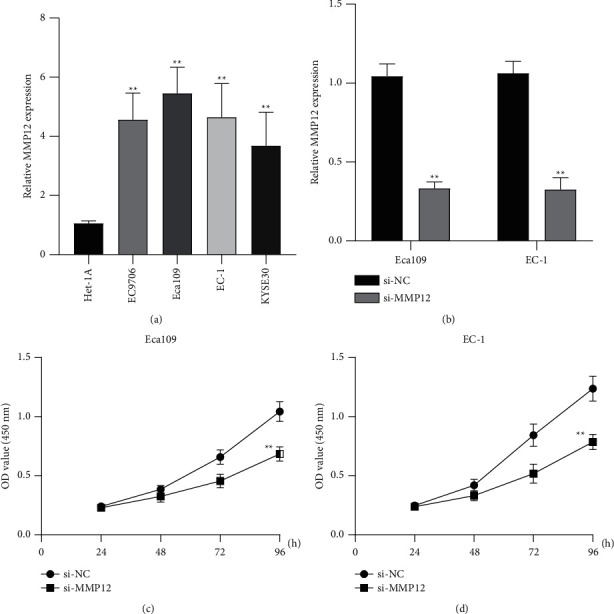
Knockdown of MMP12 suppressed the proliferation of ESCC cells. (a) RT-PCR for the expression of MMP12 in ESCC cell lines. (b) The expression of MMP12 decreased in Eca109 and EC-1 cells transfected with si-MMP12. (c), (d) CCK-8 assay indicated that OD values of Eca109 and EC-1 cells significantly decreased when transfected with si-MMP12. ^*∗∗*^*P* <  0.01.

To demonstrate the expression of MMP12 in ESCC, we performed RT-PCR using four ESCC cell lines and observed that MMP12 expression was distinctly increased in four ESCC cell lines compared with Het-1A cells (Figure 8(a)). Given that Eca109 and EC-1 exhibited a relatively higher level among four ESCC cells, we chose them for further study. We used loss-of-function experiments to explore the possible effects of MMP12 in ESCC. The interference efficiencies of siRNA are shown in Figure 8(b), suggesting that siRNA efficiently decreased MMP12 expressions. CCK-8 assays revealed that Eca109 and EC-1 proliferation was distinctly suppressed when silencing MMP12 (Figures 8(c) and 8(d)).

## 4. Discussion

The research for effective molecular markers for diagnosis and prognosis of ESCC is very important for prognosis of patients [25]. In the last decade, more and more tumor-related genes have been well studied. For instance, as a main RNA N6-adenosine methyltransferase, METTL3 was highly expressed in gastric cancer. Clinical assays disclosed that overexpression of METTL3 predicted a poor outcome of gastric cancer patients [26]. Hu et al. outlined that HIF-1*α* was distinctly enhanced on ESCC and was in line with metastasis, recurrence, and poor prognosis. Functionally, knockdown of HIF-1*α* suppressed the metastasis of ESCC cells via targeting SP1 [27]. These findings encouraged us to further identify functional genes involved in ESCC progression.

MMPs are commonly expressed in normal specimens [5]. It has been demonstrated that the expressions and activities exhibited an increased trend during inflammation and tumor progression [28, 29]. In this study, we analyzed the expressing pattern of MMPs in ESCC specimens based on TCGA datasets and identified 12 dysregulated MMPs in ESCC, including MMP1, MMP12, MMP20, MMP17, MMP14, MMP11, MMP10, MMP13, MMP9, MMP8, MMP7, and MMP3. Among the above genes, only MMP12 was associated with five-year survival of ESCC patients, and its high expression was also associated with advanced clinical stages in ESCC specimens. To explore the possible function of MMP12 in ESCC progression, we performed KEGG assays, which revealed that the genes associated with MMP12 were mainly enriched in several tumor-related pathways including PI3K-Akt signaling, estrogen signaling, and relaxin signaling [30–32]. Moreover, we also proved that knockdown of MMP12 distinctly suppressed the proliferation of ESCC cells. Besides, the effect of MMP12 has been reported in several tumors. For instance, MMP12 was highly expressed in lung adenocarcinoma, and its knockdown distinctly inhibited the growth and invasion of lung adenocarcinoma cells [33]. In hepatocellular carcinoma, high MMP12 expression predicted a poor prognosis. Importantly, the prognostic value of MMP12 was also demonstrated in 93 patients, which was consistent with our findings [34]. Thus, together with previous findings, these results indicated MMP12 as a novel prognostic biomarker for ESCC patients.

Several antibodies targeting immune checkpoints for the treatments of ESCC were approved by the FDA for the sake of its efficacy [35, 36]. Biomarkers would be tremendously valuable in improving therapeutic decision making in ESCC [37]. In this study, we elaborated on the impact of MMP12 on the immune systems. However, we did not observe a distinct different of the level of immune cells between ESCC specimens and nontumor specimens. Then, we needed to figure out whether MMP12 expression was related to immune cell on ESCC and observed that upregulated MMP12 was positively in line with the infiltration levels of macrophages M0 and mast cells activated. In addition, downregulated MMP12 was negatively correlated with the infiltration of B cells naïve, eosinophils, and mast cells resting. Our findings provided evidence that MMP12 may be a potential immunotherapeutic object for ESCC.

There are some limitations in our study. The first one is the limited sample size that needs to be improved. The second one is the lack of the exploration of mechanisms underpinning MMP12-medicated tumor immunity and the prognostic values of immune signatures. The third one is that examining the biomarkers in the serum/plasma samples might help monitor the therapy response in real-time.

## 5. Conclusion

We identified several dysregulated MMPs in ESCC, and their function needed to be further studied. We provided clinical evidence that MMP12 was highly expressed in ESCC and can serve as an independent prognostic marker for survival in ESCC. Our conclusion is that MMP12 might play a role in controlling the tumor immune microenvironments. Additional investigation is required to confirm the findings before the clinical application of MMP12.

## Data Availability

The analyzed datasets generated during the study are available from the corresponding author upon request.
